# *Bacillus anthracis* Diversity and Geographic Potential across Nigeria, Cameroon and Chad: Further Support of a Novel West African Lineage

**DOI:** 10.1371/journal.pntd.0003931

**Published:** 2015-08-20

**Authors:** Jason K. Blackburn, Moses Ode Odugbo, Matthew Van Ert, Bob O’Shea, Jocelyn Mullins, Vincent Perrenten, Angaya Maho, Martin Hugh-Jones, Ted Hadfield

**Affiliations:** 1 Spatial Epidemiology & Ecology Research Lab, Department of Geography, University of Florida, Gainesville, Florida, United States of America; 2 Emerging Pathogens Institute, University of Florida, Gainesville, Florida, United States of America; 3 Bacterial Research Division, National Veterinary Research Institute, Vom, Plateau State, Nigeria; 4 MRI Global, Palm Bay, Florida, United States of America; 5 Institute of Veterinary Bacteriology, University of Berne, Berne, Switzerland; 6 Laboratoire de Recherches Vétérinaires et Zootechniques, N’Djaména, Chad; 7 Department of Environmental Sciences, Louisiana State University, Baton Rouge, Louisiana, United States of America; Beijing Institute of Microbiology and Epidemiology, CHINA

## Abstract

Zoonoses, diseases affecting both humans and animals, can exert tremendous pressures on human and veterinary health systems, particularly in resource limited countries. Anthrax is one such zoonosis of concern and is a disease requiring greater public health attention in Nigeria. Here we describe the genetic diversity of *Bacillus anthracis* in Nigeria and compare it to Chad, Cameroon and a broader global dataset based on the multiple locus variable number tandem repeat (MLVA-25) genetic typing system. Nigerian *B*. *anthracis* isolates had identical MLVA genotypes and could only be resolved by measuring highly mutable single nucleotide repeats (SNRs). The Nigerian MLVA genotype was identical or highly genetically similar to those in the neighboring countries, confirming the strains belong to this unique West African lineage. Interestingly, sequence data from a Nigerian isolate shares the anthrose deficient genotypes previously described for strains in this region, which may be associated with vaccine evasion. Strains in this study were isolated over six decades, indicating a high level of temporal strain stability regionally. Ecological niche models were used to predict the geographic distribution of the pathogen for all three countries. We describe a west-east habitat corridor through northern Nigeria extending into Chad and Cameroon. Ecological niche models and genetic results show *B*. *anthracis* to be ecologically established in Nigeria. These findings expand our understanding of the global *B*. *anthracis* population structure and can guide regional anthrax surveillance and control planning.

## Author Summary

Anthrax, caused by the soil-borne bacterium *Bacillus anthracis*, is a disease with important public health and national security implications globally. Understanding the global genetic diversity of the pathogen is important for epidemiological and forensic investigations of anthrax events. Toward this, we describe *B*. *anthracis* genetic diversity in Nigeria and confirm it belongs to a unique West African genetic group not yet reported beyond neighboring Cameroon and Chad and Mali. This refines the global phylogeny of *B*. *anthracis*, allowing the development of more accurate diagnostics. We coupled these efforts with ecological niche modeling to map the geographic distribution of this strain group across the region. Suitable habitat for the pathogen is predicted across central Nigeria from west to east into Cameroon and Chad. Understanding the geography of *B*. *anthracis* plays an important role in informing public health by targeting disease control to high risk regions. This is particularly important in resource limited areas where intervention strategies are constrained and zoonotic disease risk is high.

## Introduction

Zoonoses, diseases affecting both human and animal populations, can exert tremendous pressures on human and veterinary health systems [1]. At the same time, many zoonoses cross the livestock/wildlife interface and have environmental reservoirs making it challenging to define at-risk areas [1], particularly in rural areas. These challenges are exacerbated in countries where public and veterinary services are financially constrained. In a recent review, some of these challenges were identified for the African country of Nigeria for several important neglected tropical diseases [2] and zoonoses [3], including brucellosis [4] and leptospirosis [5]. Anthrax, caused by the spore forming, Gram positive bacterium *Bacillus anthracis* is another zoonosis of concern in Nigeria [6,7], including a history of human cases [8].

Globally, anthrax is an important and underreported zoonosis [9] with rapid onset and high mortality in wildlife and livestock, a cause of secondary human cases, and a security risk as a bioweapon or bioterror agent [10,11]. Epidemiological or forensic investigations of anthrax benefit from the ability to subtype [12] and geo-position the pathogen [13,14]. Effective subtyping systems for the pathogen exist, including systems based on single nucleotide polymorphisms (SNPs), variable number tandem repeats (VNTRs) within a multiple locus VNTR analysis (MLVA), and single nucelotide repeat (SNRs) [15]. In particular, VNTR genotyping has described the diversity of the pathogen in numerous countries, including the United States [12,16], France [17], Georgia [18], Italy [19], Poland [20], and Kazakhstan [21]. Genotyping of larger global strain collections using expanded MLVA systems [22] and combinations of SNPs and VNTRs have added to an overall picture of *B*. *anthracis* global diversity [12].

Despite a wealth of studies addressing *B*. *anthracis* diversity globally, many regions are underrepresented and, as a result, our understanding of the worldwide population structure is incomplete. These sampling gaps not only skew descriptions of the global genetic population structure, but introduce bias in the discovery and selection of subtyping loci (e.g. canSNPs) and diagnostic markers [12,23]. For example, despite a long history of the disease on the continent, the intriguing diversity of *B*. *anthracis* strains in the western and central African countries were underrepresented in global studies [12,24] and only recently became the focus of genetic signature diversity and phylogenetic studies [22,25–27]. Lista et al. [22] subtyped strains from Cameroon using a 25-marker MLVA, assigning them to a unique “E” lineage. A later study examined the molecular diversity of *B*. *anthracis* in Chad using VNTRs and concluded the strains represented a novel and phylogenetically distinct A lineage, termed Aβ [25]. Most recently, these same authors examined bovine strains from seven sites in Cameroon and determined they were highly genetically similar to Chadian strains and were also assigned to the Aβ branch [26]. Despite these intriguing studies and unique genetics of strains from this region, the diversity of *B*. *anthracis* in the neighboring country of Nigeria has not been described.

Another key element of anthrax surveillance and control is describing the extent of the disease on the landscape and to predict at-risk areas [1,11,13]. A number of studies describe the spatial distribution of *B*. *anthracis* across the landscape using ecological niche models [11,13,28,29], including at least two studies in Africa [30,31]. These modeling approaches relate outbreak locations with non-random environmental conditions using multivariate descriptors such as temperature, soil pH, and precipitation [1]. Model outputs are binary presence/absence or probability of presence/absence on the landscape, depending on the modeling approach employed [11]. More recently, niche modeling experiments have focused on specific genetic lineages of *B*. *anthracis* to improve model accuracy [29] and to explore genetic/ecological differences between related strains within lineages [32]. Such models provide insights into the ecological conditions associated with the disease and areas of pathogen persistence. Species’ range predictions provide first estimates of at-risk areas to identify passive surveillance priorities. Additionally, these modeling approaches can be coupled with spatial clustering techniques to better define livestock outbreak hotspots and these maps can direct priorities for active control, such as vaccination [33].

In this study, we use VNTR [22] and SNR [34] typing systems, and associated public databases, to describe strains from Nigeria. The aim of our study was to examine the genetic diversity of *B*. *anthracis* in Nigeria, map the genotype data with published strain data from bordering countries, and produce an ecological niche model-based prediction of the potential distribution of *B*. *anthracis* across Cameroon, Chad, and Nigeria.

## Materials and Methods

### Organisms/DNA Extraction

Five archival cultures of *B*. *anthracis* isolated from domestic cattle were provided by the National Veterinary Research Institute, Plateau State, Nigeria for analysis. Cultures were grown on sheep blood agar and checked for purity. One culture failed to grow; remaining viable cultures were evaluated for phage sensitivity and DNA was extracted for polymerase chain reaction (PCR) reactivity for lethal factor, *capB* and a chromosomal marker (Table 1) following the protocol of [16]. Cells were harvested from sheep blood agar and DNA was extracted using the Qiagen DNeasy kit (Qiagen, Germantown, Maryland, USA) [16]. Resulting DNA was filter sterilized using a 0.22 μL spin filter. Sterile DNA samples were used for genotyping. Sterile DNA of Chadian isolates described by Maho et al. [25] were provided by V. Perrenten.

**Table 1:** Gene targets and primers for polymerase chain reaction (PCR) to detect *Bacillus anthracis* used in this study.

Gene target	Forward primer	Reverse primer	Probe
lethal factor	CACTATCAACACTGGAGCGATTCT	AATTATGTCATCTTTCTTTGGCTCAA	6FAM- AGCTGCAGATTCC-BHQ
capB	TAAGCCTGCGTTCTTCGTAAATG	GTTCCCAAATACGTAATGTTGATGAG	6FAM- TTGCAGCGAATGAT-BHQ
BA-1 chromosome	GTACATCTTCTAGCTGTTGCAA	ACGTAGGAAGACCGTTGATTA	6FAM- CGTTGTTGTGTATTTG-BHQ

### MLVA and SNR Genotyping

Genetic diversity of *B*. *anthracis* was evaluated using the 25 marker MLVA described by Lista et al. [22], with slight modification. Specifically, we adapted the MLVA-25 to a 5-dye fluorescent chemistry on an Applied Biosystems 3100 Genetic Analyzer platform. Data analysis was done using GeneMapper software (Applied Biosystems, Foster City, California, USA). MLVA-25 results from Nigerian and Chadian isolates were normalized to published values from Lista et al. [22] by comparison to reference alleles from common and shared laboratory strains. We examined genetic relationships among the global strains using Unweighted Pair Group Method with Arithmetic Averages (UPGMA) clustering analysis of the VNTR data set and the published VNTR data of Lista et al. [22]. Distance matrices were generated in PAUP 4.0 [35] and tree robustness was evaluated by performing a bootstrap analysis with 100 replicates. Distance matrices were then exported into the MEGA 6.0 software package [36] for dendrogram construction. SNR-4 typing of Nigerian strains was performed as described by Kenefic et al. [34], however, due to low yields on HM-2 in multiplex reactions and to confirm SNR differences, we repeated all samples and loci in 10 μL single primer reactions containing 0.2 μm of each primer,0.5 U pfu Turbo Polymerase, 1 mM dNTPs, 1X PCR buffer, and 3mM MgCl_2_ (supplemental MgCl_2_ added to 2mM level in 10X buffer).

### Ecological Niche Modeling

**Fig 1:**
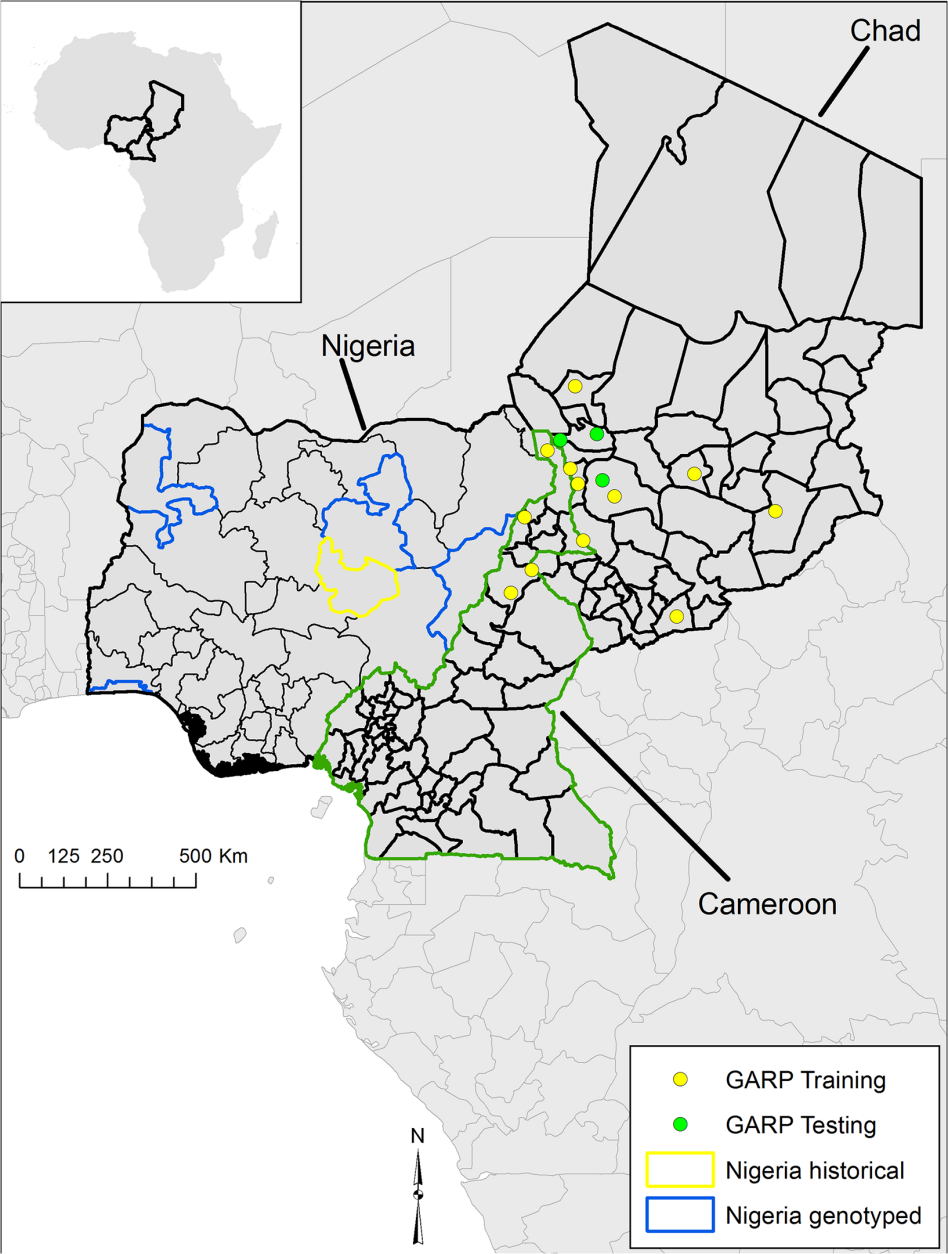
The geographic distribution of *Bacillus anthracis* isolates by prefecture for Nigeria and by village of isolation for, Chad, and Cameroon. Nigerian isolates and source locations are first reported herein this study. Locations for Chad were reported by Maho et al. [25] and Cameroonian isolates were reported by Pilo et al. [26]. Points represent those used to build train (yellow points) and test (green points) ecological niche models using GARP on a combined native landscape of Cameroon and Chad. Those models were projected on to Nigeria to compare with historical regions reporting disease.

Spatial data on all five Nigerian isolates were provided at the prefecture level (a sub-national political unit; Fig 1) by The Nigerian National Veterinary Research Institute, meaning they could not be used in ecological niche models. Spatial data, provided as village names, for isolates from Chad were extracted from Maho et al. [25]. Cameroonian village names of isolates were extracted from Pilo et al. [26]. Village latitude and longitude coordinate pairs were derived from the National Geospatial Intelligence Agency GEOnet names server, *Index Mundi*, and the GeoNames database following Kracalik et al. [37] and mapped using ArcGIS 10.1 (ESRI, Redlands, California, USA) (Fig 1). These data were used as species occurrence data (point locations) for ecological niche modeling experiments.

We built ecological niche models using spatially unique locations of *B*. *anthracis* isolates from Cameroon and Chad as a single landscape. The genetic algorithm for rule-set prediction (GARP) ecological niche modeling tool was used to construct all models. Briefly, GARP is a presence-only genetic algorithm that models species’ potential geographic distributions through an iterative process of training and testing that occurs through resampling and replacement of input data [38]. Species’ occurrence data, i.e. village locations of confirmed *B*. *anthracis* isolates from Cameroon and Chad, and ecological data from global gridded datasets of climatic variables [39,40] were input into the model as latitude/longitude coordinate pairs and ascii raster files, respectively. A pattern matching process was applied to evaluate non-random relationships between species localities and ecological variable combinations derived through iteration in a genetic algorithm. These relationships are then written as a series of if/then logic statements (known as rules) to define species’ presence or absence. The modeling process is two-step, first building rules in variable space and then projecting rules onto the landscape to predict the potential distribution of the species [41]. Because spatial data suitable for model building were unavailable for Nigeria, we opted to build models of Cameroon and Chad as a single landscape. As a result of the two-step process described above, rule-sets can be projected or transferred onto novel landscapes using a comparable ecological dataset [1]. In this study, we built models for Cameroon and Chad and transferred those models onto the Nigerian landscape to estimate the geographic potential of the pathogen in that country.

All models were produced in DesktopGARP version 1.1.3 [DG] and maps were generated using ArcGIS 10.1. As described elsewhere [13], we used a modeling experiment with 200 models using a convergence threshold of 0.01, 1000 maximum iterations, and a best subset procedure selecting for the 10 best models based on a 10% omission threshold and 50% commission [42]. To be compatible with other *B*. *anthracis* modeling efforts [28], a set of previously defined environmental variables describing elevation and measures of temperature, precipitation, and the normalized difference vegetation index was used (Table 2). Environmental covariates are described in detail elsewhere [39,40]. For this study, we used 8x8 km (0.10 decimal degree) gridded environmental data. Given the limited sample size of spatial locations available for modeling, and a lack of data from Nigeria, we took a conservative approach to model development and interpretation. For modeling experiments, we performed 10 random training/testing splits of the Cameroonian/Chadian combined data and projected onto Nigeria each time. We then extracted those portions of each experiment where all 10 models in the best subset were in agreement and summated those across all 10 model experiments. In this way, any pixel mapped as suitable habitat on either the combined Cameroonian/Chadian landscape or Nigeria were predicted by at least 10 models in a best subset. Therefore, those areas predicted by all 10 experiments represent pixels predicted by 100 models meeting the best subset criteria.

**Table 2:** Environmental variables used to construct ecological niche models for Cameroon and Chad as a single landscape and project the potential distribution of *Bacillus anthracis* in Nigeria.

Environmental Variables	Name	Source
Annual Mean Temperature	BIO1	Hijmans et al. [39]
Temperature Annual Range	BIO7	Hijmans et al. [39]
Annual Precipitation	BIO12	Hijmans et al. [39]
Precipitation of Wettest Month	BIO13	Hijmans et al. [39]
Precipitation of Driest Month	BIO14	Hijmans et al. [39]
Elevation (Altitude)	ALT	Hijmans et al. [39]
Mean Annual NDVI	wd1014a0	Hay et al. [40]
Annual NDVI Amplitude	wd1014a1	Hay et al. [40]

We used the area under the curve (AUC) in a receiver operating characteristic (ROC) analysis [43] to evaluate model accuracy for the combined Cameroonian/Chadian models following Joyner et al. [28]. While a metric with known limitations [44], AUC scores provide a measure of model accuracy using testing data withheld from the model training process. We used AUC, total omission, and the summed area omission (SAO) [32] metrics together to evaluate niche models. To evaluate Nigeria, we compared predictions to the prefectures assigned to historical isolates.

## Results

While limited to four samples for genotyping, the available Nigerian samples were all isolated from domestic cattle and broadly geographically distributed across the country with each from separate prefectures (Fig 1). Chadian isolates were distributed across five prefectures in the southern part of the country along the Nigerian and Cameroonian borders. All Nigerian isolates were collected and archived from 1949 to 1966. In contrast, the nine Chadian strains were collected from 1996 to 2003. Dates of isolation were not reported for the Cameroonian strains in Lista et al. [22], but the more recent cattle strains from the country were isolated between 1988 and 2004.

**Fig 2:**
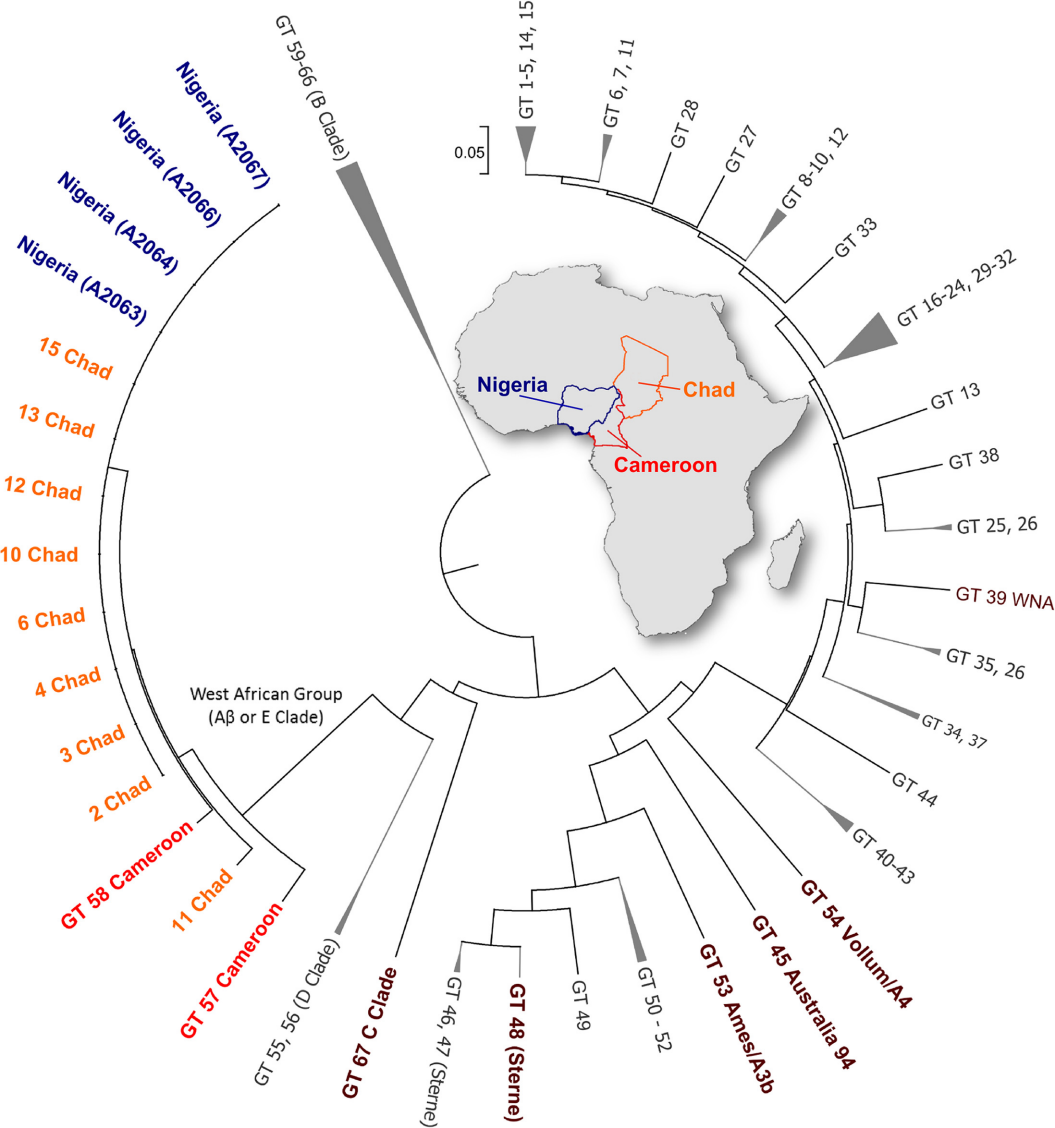
Dendrogram based on MLVA of 25 markers of Nigerian, Chadian *B*. *anthracis* strains and the 67 genotypes reported by Lista et al. [22], including Cameroon. The dendrogram was generated using Unweighted Pair Group Method with Arithmetic Mean (UPGMA) clustering.

MLVA-25 subtyping of the Nigerian, Chadian and Cameroonian isolates indicated they were closely related genetically; differing only in one or two VNTR loci (Table 3). UPGMA cluster analysis with the 67 published genotypes [22], indicate these isolates form a unique clade in the tree (Fig 2). In two independent analyses the bootstrap support for the branch was 100, a value which exceeded support for the widely recognized B clade (bootstrap value = 98). Further analysis of the Nigerian strains using 4 highly mutable loci revealed 4 unique SNR sub-types. Clear single base mutational changes were detected in the HM-1 and HM-6 loci. The HM-2 locus was excluded from the analysis due to consistently weak amplification (in both single and multiplex reactions) and a very complex and difficult to interpret ‘fingerprint’ pattern. The SNR-4 data are reported in Table 4.

**Table 3:** MLVA-25 repeats for samples from Nigeria, Chad, and Cameroon available for this study. Data from Cameroon were presented in Lista et al. [22]. Bold letters indicate repeat differences between isolates within that locus.

Country/Strain	Town/ village	Region/State	*vrrA*	*vrrB*_1_	*vrrB*_2_	*vrrC*_1_	*vrrC*_2_	CG3	pXO1	pXO2	Bam1	Bam3	Bam5	Bam13	Bam15	Bam21	Bam22	Bam23	Bam24	Bam25		Bam30	Bam31	Bam34	Bam44	Bam51	Bam53
Chad 2	N’djaména	Chari Baguirmi	8	16	8	57	21	2	6	6	13	21	6	23	43	10	11	10.5	8	13	14	-1	-1	9	6	9	8
Chad 3	Massakory	Chari Baguirmi	8	16	8	57	21	2	6	6	13	21	6	23	43	10	11	10.5	8	13	14	-1	-1	9	6	9	8
Chad 4	Dourbali	Chari Baguirmi	8	16	8	57	21	2	6	6	13	21	6	23	43	10	11	10.5	8	13	14	-1	-1	9	6	9	8
Chad 6	Am-Timan	Salamat	8	16	8	57	21	2	6	6	13	21	6	23	43	10	11	10.5	8	13	14	-1	-1	9	6	9	8
Chad 10	Bitkine	Guera	8	16	8	57	21	2	6	6	13	21	6	23	43	10	11	10.5	8	13	14	-1	-1	9	6	9	8
Chad 11	Mandelia	Chari Baguirmi	8	16	8	57	21	2	6	6	13	21	6	23	43	10	11	10.5	8	13	14	-1	-1	9	6	**10**	8
Chad 12	Bongor	Mayo Kebbi	8	16	8	57	21	2	6	6	13	21	6	23	43	10	11	10.5	8	13	14	-1	-1	9	6	9	8
Chad 13	Moîssala	Moyen-Chari	8	16	8	57	21	2	6	6	13	21	6	23	43	10	11	10.5	8	13	14	-1	-1	9	6	9	8
Chad 15	Karal	Chari Baguirmi	8	16	8	57	21	2	6	6	13	21	6	23	43	10	11	10.5	8	13	14	-1	-1	9	6	9	8
Nigeria 1555	N/A	Adamwa	8	16	8	57	21	2	6	6	13	21	6	23	43	10	11	10.5	8	13	14	60	64	9	6	9	8
Nigeria 829	N/A	Plateau	8	16	8	57	21	2	6	6	13	21	6	23	43	10	11	10.5	8	13	14	60	64	9	6	9	8
Nigeria Gwandu	N/A	Kebbi	8	16	8	57	21	2	6	6	13	21	6	23	43	10	11	10.5	8	13	14	60	64	9	6	9	8
Nigeria Lagos	N/A	Lagos	8	16	8	57	21	2	6	6	13	21	6	23	43	10	11	10.5	8	13	14	60	64	9	6	9	8
Cameroon L58	N/A	N/A	8	16	8	57	21	2	6	6	13	21	6	24	43	10	11	10.5	8	13	14	60	64	9	6	9	8
Cameroon L57	N/A	N/A	8	16	8	57	21	2	7	6	13	21	6	**33**	43	10	11	10.5	8	13	14	60	64	9	6	9	8

**Table 4:** SNR-4 allele sizes for *Bacillus anthracis* isolates from Nigeria. Sizes are rounded to the nearest base pair. See S1 Fig Information for actual electrophoresis data.

Sample	HM-1	HM-2	HM-6	HM-13
UF1052	85	Excluded	88	115
UF1062	83	Excluded	87	115
UF1063	84	Excluded	87	115
UF1075	85	Excluded	87	115

**Fig 3:**
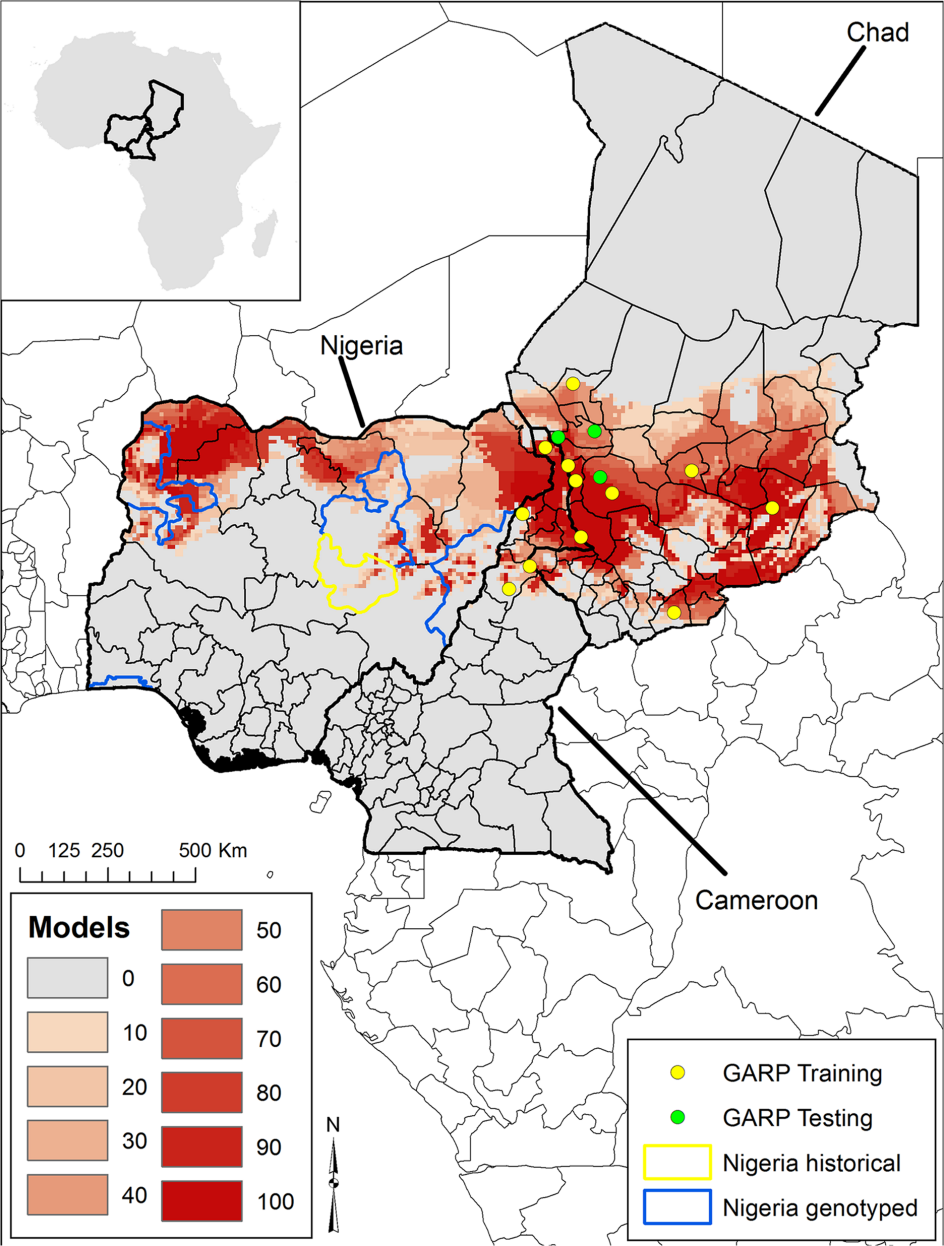
Ecological niche modeling-based predictions of *Bacillus anthracis* in Cameroon and Chad projected onto Nigeria (Inset illustrates the location of these countries within Africa). Here we illustrate the summation of only the region of model agreement of all 10 models within 10-model best subsets across all five modeling experiments. The color ramp from light to dark red indicates the number of models in agreement from 10 GARP modeling experiments.

All GARP experiments reached convergence prior to 1,000 iterations. Across the 10 experiments, the average AUC was 0.71 (range 0.57–0.86) and all AUC values were statistically significant, indicating these experiments predicted better than random. Across experiments, the best subsets predicted at least 1 of the 3 the external testing points, with 8 of the 10 experiments predicting 100% (3/3) of the testing data. The summated GARP best subsets suggest a band of suitable habitat across northern Cameroon and southern Chad east to the Nigerian border (Fig 3). Generally there was broad agreement between the 10 best model subsets (red areas), particularly where the two countries meet Nigeria’s eastern border. Projected models suggest a broad band of suitable habitat across northern Nigeria from east to west, with areas of greatest model overlap in the northeast and northwest. GARP predicted some portion of the landscape in each of four interior prefectures reported historically, excluding only the coastal report.

## Discussion

This study is the first to describe the genetic diversity of *B*. *anthracis* associated with historical cattle outbreaks in Nigeria, Africa. Although the number of isolates tested was low, the genetic similarity of isolates within the country, coupled with their wide geographic distribution and long temporal separation suggest the strains are ecologically established and genetically stable. From a broader regional perspective, the Nigerian MLVA genotypes are highly similar to isolates from Chad [25] and the previously described Cameroonian isolates; described alternatively as belonging to the Aβ lineage [25,26] and the E group [22]. Tamborrini et al. [27] reported Mali strains may also be in this lineage based on immunological and genetic analyses. Our report of this strain group in Nigeria confirms the lineage is widespread in cattle in this West African region and expands its geographic extent. Further, strains in this study were isolated over 6 decades indicating a high level of temporal strain stability. This finding is particularly intriguing since the report of Tamborrini et al. [27] describes this unique genetic lineage as anthrose deficient, unlike most other *B*. *anthracis* lineages. The authors’ findings show the Sterne vaccine elicits a strong immunologic response to anthrose in cattle. When coupled with high livestock anthrax incidence across the region [27], this lead to the hypothesis that the anthrose deficient strain group represents a vaccine escape mutant. Our sequence data from a representative Nigerian strain confirm the same anthrose deficient genotypes reported by Tamborrini et al. [27], with Nigeria having both the 8 base pair frameshift mutation in the aminotransferase gene (BAS3320; S2 Fig) and the SNP in the glycosyltransferase gene (BAS3321) that together result in premature stop codons. Therefore, our data suggest this escape mutation was present at least 60 years ago; a timeframe consistent with the mass introduction of the Sterne vaccine across the continent. Our sample size was limited and almost certainly underrepresents diversity within Nigeria. Without access to additional samples, including isolates from more recent outbreaks in the country we cannot determine if this is the dominant lineage present in Nigeria. However, the larger sample sizes from Chad reflecting more recent isolations of this lineage suggest it is actively circulating in the region.

The trans-border genetic relatedness of *B*. *anthracis* across the region may be a consequence of historic trade patterns and continued nomadic pastoralism [45], which function to disseminate a fit strain complex. This strain group has not been observed outside this region, indicating some level of niche specialization. There is also an absence of trans-global strain groups (e.g. Trans-Eurasian A sub-group [12]), which argues against recent foreign introductions of anthrax strains into these countries. This may be a reflection of the isolation of these areas and the nature of commerce favoring intra-regional trade. The geographic extent of this genetic lineage can only be approximated until additional isolates are obtained and genotyped. Interestingly, none of the four available Nigerian strains resembled the intriguing *B*. *cereus* anthrax causing organisms previously reported in primates in West Africa [46] and more recently from livestock in Cameroon [26]. However, this may simply be a function of under sampling and underreporting.

The spatial distribution defined by our ecological niche modeling experiments suggest a west to east band of suitable habitat for *B*. *anthracis* across all three countries with many of the historical outbreaks from Cameroon and Chad predicted by the models (Fig 3). Although there are limited historical reports on the distribution of anthrax for Nigeria, our models predicted much of north central Nigeria, where large epizootics were previously reported [7]. Additionally, this area encompasses the northern core states of Nigeria, which historically provided much of the countries meat supply [47]. Much of this meat production comes from the large population of nomadic Fulani’s; a group with limited access to modern veterinary medicine [47]. One of our isolates was from southern Nigeria along the coast and outside of our predicted suitability. In at least one other historical report, anthrax affected captive carnivores in Ibadan, Nigeria, where the suspected source was contaminated meat [47]. Other earlier studies describing anthrax outside of our predicted areas reported sporadic and occupational anthrax (e.g. meat handling) [6]. While our estimates for Nigeria are limited to model projections, the agreement between predicted areas and the prefectures where the organisms were isolated, coupled with contemporary spatial data for Chad and Cameroon, suggest models identify *B*. *anthacis* suitable habitat for Nigeria. These maps serve as a first estimate of the geographic distribution for this pathogen and this unique lineage and are informative for prioritizing surveillance regionally. However, our models are limited to *B*. *anthracis* strains in this West African Group and may underestimate the overall distribution of these three countries if other strains/lineages are present [29].

In summary, our findings support recent reports that West Central African cattle strains are dominated by the phylogenetically unique West African strain group and extend the boundaries of the group across Nigeria. Considering the geographic extent, stability and uniqueness of this strain complex, further genomic studies should be conducted to more accurately position these strains into the global evolutionary history of *B*. *anthracis*. Our ecological niche modeling experiments provide a starting point for estimating anthrax risk in the region and specifically Nigeria.

## Supporting Information

S1 Fig SNR. Electropherogram overlay illustrating the different SNR alleles detected across Nigerian isolates.
(PNG)

S2 Fig Sequence alignment. Sequence alignment of the Nigeria strain 1555 anthrose biosynthesis aminotransferase gene (1,139 base pair, BAS3320) with several representative *B*. *anthracis* strains. The 8-nucleotide tandem repeat AAAAAAAG is present in 2 copies in the majority of global strains, with the 3 copy polymorphism present in Nigerian and West African strains and associated with the anthrose deficient phenotype (Tamborrini et al, 2011).
(TIF)
