# Correction: *Bacillus anthracis* Diversity and Geographic Potential across Nigeria, Cameroon and Chad: Further Support of a Novel West African Lineage

**DOI:** 10.1371/journal.pntd.0004089

**Published:** 2015-09-29

**Authors:** Jason K. Blackburn, Moses Ode Odugbo, Matthew Van Ert, Bob O’Shea, Jocelyn Mullins, Vincent Perreten, Angaya Maho, Martin Hugh-Jones, Ted Hadfield

The sixth author’s name is spelled incorrectly. The correct spelling is: Vincent Perreten. The correct citation is: Blackburn JK, Odugbo MO, Van Ert M, O’Shea B, Mullins J, Perreten V, et al. (2015) *Bacillus anthracis* Diversity and Geographic Potential across Nigeria, Cameroon and Chad: Further Support of a Novel West African Lineage. PLoS Negl Trop Dis 9(8): e0003931. doi:10.1371/journal.pntd.0003931
