# Supplementary figures and images for: Bacillus anthracis Diversity and Geographic Potential across Nigeria, Cameroon and Chad: Further Support of a Novel West African Lineage
Source: PLoS Negl Trop Dis. 2015 Aug 20;9(8):e0003931. doi: 10.1371/journal.pntd.0003931 (PMC4546381; doi:10.1371/journal.pntd.0003931)

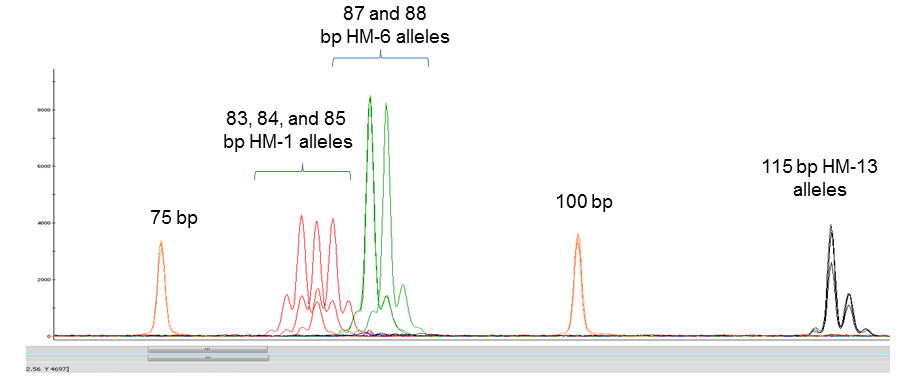

Supplement: S1 Fig — Electropherogram overlay illustrating the different SNR alleles detected across Nigerian isolates. (PNG) [file pntd.0003931.s001.png]

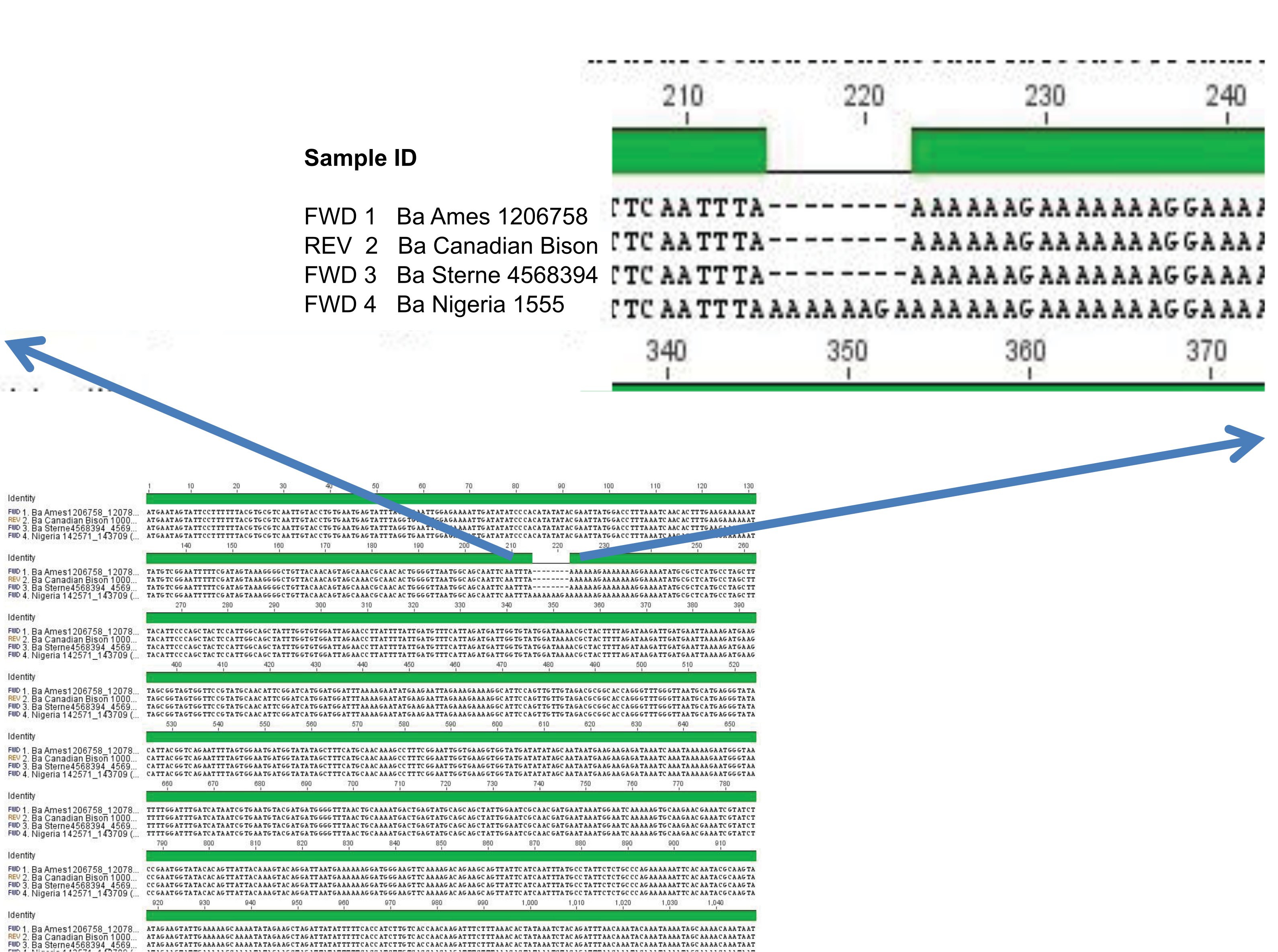

Supplement: S2 Fig — Sequence alignment of the Nigeria strain 1555 anthrose biosynthesis aminotransferase gene (1,139 base pair, BAS3320) with several representative B. anthracis strains. The 8-nucleotide tandem repeat AAAAAAAG is present in 2 copies in the majority of global strains, with the 3 copy polymorphism present in Nigerian and West African strains and associated with the anthrose deficient phenotype (Tamborrini et al, 2011). (TIF) [file pntd.0003931.s002.tif]
